# Quantitative assessment of lung opacities from CT of pulmonary artery imaging data in COVID-19 patients: artificial intelligence versus radiologist

**DOI:** 10.1093/bjro/tzaf008

**Published:** 2025-04-29

**Authors:** Ann Mari Svensson, Anna Kistner, Kristina Kairaitis, G Kim Prisk, Catherine Farrow, Terence Amis, Peter D Wagner, Atul Malhotra, Piotr Harbut

**Affiliations:** Department of Molecular Medicine and Surgery, Karolinska Institutet, Stockholm, 171 76, Sweden; Department of Radiology, Solna, Karolinska University Hospital, Stockholm, 171 76, Sweden; Department of Molecular Medicine and Surgery, Karolinska Institutet, Stockholm, 171 76, Sweden; Medical Radiation Physics and Nuclear Medicine, Imaging and Physiology, Solna, Karolinska University Hospital, Stockholm, 171 76, Sweden; Ludwig Engel Centre for Respiratory Research, Westmead Institute for Medical Research, Sydney, NSW 2145, Australia; Department of Respiratory and Sleep Medicine, Westmead Hospital, University of Sydney, Sydney, NSW 2145, Australia; Department of Medicine, University of California, San Diego, CA 92037, United States; Ludwig Engel Centre for Respiratory Research, Westmead Institute for Medical Research, Sydney, NSW 2145, Australia; Department of Respiratory and Sleep Medicine, Westmead Hospital, University of Sydney, Sydney, NSW 2145, Australia; Ludwig Engel Centre for Respiratory Research, Westmead Institute for Medical Research, Sydney, NSW 2145, Australia; Department of Medicine, University of California, San Diego, CA 92037, United States; Department of Medicine, University of California, San Diego, CA 92037, United States; Department of Medical Sciences, Danderyd Hospital, Karolinska Institutet, Stockholm, 182 88, Sweden

**Keywords:** lung opacities in COVID-19, AI analyses compared to the radiologist, radiological contrast medium and lung opacities

## Abstract

**Objectives:**

Artificial intelligence (AI) deep learning algorithms trained on non-contrast CT scans effectively detect and quantify acute COVID-19 lung involvement. Our study explored whether radiological contrast affects the accuracy of AI-measured lung opacities, potentially impacting clinical decisions. We compared lung opacity measurements from AI software with visual assessments by radiologists using CT pulmonary angiography (CTPA) images of early-stage COVID-19 patients.

**Methods:**

This prospective single-centre study included 18 COVID-19 patients who underwent CTPA due to suspected pulmonary embolism. Patient demographics, clinical data, and 30-day and 90-day mortality were recorded. AI tool (Pulmonary Density Plug-in, AI-Rad Companion Chest CT, SyngoVia; Siemens Healthineers, Forchheim, Germany) was used to estimate the quantity of opacities. Visual quantitative assessments were performed independently by 2 radiologists.

**Results:**

There was a positive correlation between radiologist estimations (*r*^2^ = 0.57) and between the AI data and the mean of the radiologists’ estimations (*r*^2^ = 0.70). Bland-Altman plot analysis showed a mean bias of +3.06% between radiologists and −1.32% between the mean radiologist vs AI, with no outliers outside 2×SD for respective comparison.

The AI protocol facilitated a quantitative assessment of lung opacities and showed a strong correlation with data obtained from 2 independent radiologists, demonstrating its potential as a complementary tool in clinical practice.

**Conclusion:**

In assessing COVID-19 lung opacities in CTPA images, AI tools trained on non-contrast images, provide comparable results to visual assessments by radiologists.

**Advances in knowledge:**

The Pulmonary Density Plug-in enables quantitative analysis of lung opacities in COVID-19 patients using contrast-enhanced CT images, potentially streamlining clinical workflows and supporting timely decision-making.

## Introduction

The rapid advancement of artificial intelligence (AI) in medical imaging has transformed disease diagnosis and management, including COVID-19. CT pulmonary angiography (CTPA) is vital for assessing severe complications like pulmonary embolism (PE). While AI effectively analyses non-contrast CT scans for lung opacities, its use in contrast-enhanced CTPA images remains untested.

CT findings in COVID-19 lungs typically include bilateral opacities with a peripheral, posterior, and basal distribution, appearing as subpleural ground-glass opacities (GGO) in early stages.^1–3^ These can progress to widespread consolidations and patterns suggestive of organizing pneumonia (OP), such as perilobular opacities, nodules, or reverse halo appearances.^4^^,^^5^ Severe cases may exhibit a crazy paving (CP) pattern, indicating extensive lung involvement and potential acute respiratory distress syndrome (ARDS).^6^^,^^7^

AI and deep learning algorithms on non-contrast CT scans outperform traditional radiomics and manual assessments in detecting and quantifying COVID-19 severity.^8^^,^^9^ In acute phases, combining clinical data with opacity metrics enable accurate short-term outcome predictions.^10^

During the pandemic’s first wave, high 60-day mortality was linked to widespread opacities combined with PE.^11^^,^^12^ Clinicians often opt for CTPA over non-contrast CT when thromboembolism is suspected, but this may hinder AI-trained quantification of lung opacities due to the use of contrast. Extending AI’s capabilities to analyse opacities on CTPA images, alongside detecting PE, would be clinically valuable. Our study aimed to evaluate the efficacy of an AI algorithm in analysing parenchymal opacities on CTPA scans.

We hypothesized that the presence of radiological contrast, by influencing the lung opacity measurements conducted by a deep learning algorithm trained on images without contrast enhancement, may potentially lead to artificially elevated results for the quantity of the parenchymal lung changes. To examine this possibility, we compared the extent of lung opacity as determined by commercially available AI software against the visual assessments performed by 2 experienced radiologists. Our analysis utilized CTPA images from patients in the early stages of COVID-19.

## Methods

### Study design

This prospective single-center study enrolled 18 acute-phase COVID-19 patients at Danderyds University Hospital, Stockholm, from November 1 to December 15, 2020, following ethical approval (Swedish Ethical Review Authority, application 2020-02966) and written informed consent. Data collection and analysis were conducted under a collaboration between Region Stockholm and the University of California, San Diego. None of the patients had a diagnosis indicating pre-existing lung disease at the time of COVID-19 diagnosis. The included patients had a confirmed positive reverse transcription polymerase chain reaction test for SARS-CoV-2. Patients were referred for CTPA due to suspected PE.

In our study, 2 CT scanners were used: the dual-source SOMATOM Drive and the single-source SOMATOM Edge Plus, both Siemens Healthineers, Forchheim, Germany. The SOMATOM Drive operated with Flash mode, resulting in a pitch value of 2.2 compared to SOMATOM Edge Plus, which had a pitch of 1.2. Automated tube current modulation (CareDose4D) and automatic tube voltage selection (Care kV) were employed to optimize imaging, which introduce slight variability in kV values depending on the patient's size and body composition. These parameters were selected to ensure optimal contrast and minimize radiation exposure, maintaining consistency across scans as much as possible. Intravenous injection of 31-72 mL iodinated contrast agent (Iomeron 400 mg/mL) with a flow rate of 3.6-6.0 ml/s was used. The dose-length product ranged from 73 to 331 milligray* cm, and the CT dose-index volume ranged from 2.6-11.7 milligray* cm. Detailed CT scan acquisition data are provided in Appendix S1.

Information regarding the clinical course of the disease and comorbidities was collected, as were demographics, including age, sex, weight, length, body mass index, and 30-day and 90-day mortality post-hospital discharge.

### CTPA image analysis

The presence of pulmonary emboli was independently analysed by 2 radiologists on a PACS workstation (SECTRA AB), employing 0.63-mm slice reformats in orthogonal planes [3].

The pulmonary density plug-in (AI-Rad Companion Chest CT SyngoVia; Siemens Healthineers, Erlangen, Germany), an AI tool, was used to estimate the percentage of opacity distribution, lung volumes, and volume of opacities. The algorithm can then segment lesions, lungs, and lobes in 3 dimensions and provide a comprehensive measure for assessing the severity of pulmonary parenchymal involvement.

In our study, thin-section CT images were processed by the AI tool to obtain quantitative features, including lung volumes, percentage of all-type and high-attenuation opacities (≥ −200 Hounsfield Units [HU]), mean HU, and SD of opacities within a given lung region.^13^ The areas of interest for the opacity calculations were chosen according to the automated segmentation protocol which is a feature of the AI tool used. The percentage of opacity analysed is calculated as the total percent volume of the lung parenchyma that is affected by disease with the quantification difference threshold of 1%.

Two experienced radiology consultants, independently assessed COVID-19-specific pulmonary opacities across axial, coronal, and sagittal planes without access to AI results. They estimated visually increased lung opacities, from ground-glass to consolidations, in 5% increments of total lung volume, including even the changes of other aetiology. Other findings, such as OP, atelectasis, bronchial dilatation, and pleural effusion, were also recorded. Using a window centre of −400 and width of 1600, the radiologists ensured optimal contrast to detect subtle changes. This systematic approach, based on established frameworks adapted for COVID-19, ensured accurate and consistent evaluation of lung involvement.^14^^,^^15^

### Statistical analysis

Demographic and clinical data have been presented as medians and ranges, with the radiological data being presented as mean (95% CI). Linear regression analysis was performed to compare the 2 radiologists’ assessment percentage opacities as well as the mean values obtained by the radiologists and the AI data. For comparison of the 2 radiologists’ quantification of the percentage of opacities as well as the mean values obtained by the radiologists and the AI data Bland-Altman plots have been drawn and analysed.

The model’s performance was evaluated using receiver operating characteristic (ROC) analysis and area under the curve (AUC) calculations. Using the mean radiologist assessment as the reference standard, a 5% interval was set as the criterion for a positive match between the AI’s predictions and the radiologist's evaluation. ROC analysis was conducted at thresholds of 10%, 20%, 30%, 40%, and 50%. Sensitivity and specificity values were calculated for each threshold to assess the system's performance comprehensively.

A *P* < .05 was considered significant. Statistical analyses were performed using statistical software Stat Soft, version 1 (Tulsa, OK, USA) and DataGraph V 5.2 (Visual Data Tools, Chapel Hill, NC, USA).

## Results

### General patient data

General patient data are summarized in Table 1. The study population consisted of 18 mostly middle-aged, obese patients, 78% of whom were men. Ten patients received supplementary oxygen treatment. Fifteen patients were hospitalized for a duration ranging between 2 and 12 days. The other 3 were not considered in need of hospital admission and were discharged directly from the emergency room. None of the patients died during the follow-up period of up to 90 days.

**Table 1.** General data.

Demographics	Patients (*n* = 18)
Gender (male/female) (no.)	14/4
Age (years)	50 (31-54)
Weight (kg)	93 (74-100)
Height (cm)	181 (172-186)
BMI (kg/m^2^)	26.6 (25.2-30.8)
**Comorbidities and outcome**	
Time from symptoms to CT (days)	10 (7-13)
Total hospitalization (days) (*n* = 15)	4 (2-12)
Hypertension (%) (no. yes/no)	11 (2/16)
Hyperlipidemia (%) (no. yes/no)	11 (2/16)
Smoking (%) (no. yes/no)	17 (3/15)
Cardiovascular (%) (no. yes/no)	6 (1/17)
Diabetes (%) (no. yes/no)	11 (2/16)
COPD (%) (no. yes/no)	6 (1/17)
Asthma (%) (no. yes/no)	17 (3/15)
Other lung diseases (%) (no. yes/no)	0 (0/18)
Hospitalized after CT (%) (no. yes/no)	83 (15/3)
30-/90-day mortality (no.)	0/0
**Supplemental oxygen at CTPA**	
Need of supplementary oxygen yes/no (no.)	10/8
FIO_2_	25 (21-45)

The table summarizes patient cohorts’ demographics as well as comorbidities, outcome data, and need of supplemental oxygen at the time of CTPA. Values are presented as median (range) unless stated otherwise.

Abbreviations: BMI = body mass index; COPD = chronic obstructive pulmonary disease; CTPA = CT pulmonary angiography.

### Descriptive CT findings

PE was not detected in any of the CTPA examinations performed. Descriptive CT findings with definitions are presented in detail in Appendix S2. All patients had GGO, and all but one had consolidations. The perilobular pattern, often accompanied by parenchymal bands, was the most common sign of OP, primarily seen in the lower zones. The atoll sign appeared in 1 patient.

CP was less common, typically occurring alongside OP, and only 2 cases suggested possible ARDS or diffuse alveolar damage. Minor atelectasis was occasionally observed as part of OP and was never lobar. In 2 cases, vertebral osteophytes caused localized atelectasis unrelated to COVID-19.

Bronchial dilatation occurred in 50% of cases, limited to lower lobes, and pleural effusions were minimal (<2 cm). No dense fibrosis was observed, consistent with the study’s acute phase focus. No patient met the definition of irreversible fibrosis.^16^

Nodules were recorded as part of OP, with no other nodules or calcifications noted. Nearly all patients had bilateral, multilobar changes, predominantly peripheral and in the lower zones.

### Opacity quantification

Radiological findings and opacity data are summarized in Table 2.

**Table 2.** Opacity quantification.

Radiologic findings—CTPA	**Patients** (*n* = 18)
Pulmonary embolism (%)	0
**AI estimation of opacity**	
Percentage opacity (%)	34.8 (28-42)
**Radiologists’ estimation of opacity**	
Radiologist no 1 opacity percentage	35.0 (28-42)
Radiologist no 2 opacity percentage	31.9 (24-40)
Mean radiologist opacity percentage	33.5 (26-41)

Measurements done by AI and radiologists. Pulmonary embolism assessed by the attending radiologist. Values are presented as mean (±95% CI).

Abbreviation: CTPA = CT pulmonary angiography.

#### Linear regression and correlation analysis of lung opacity quantitative estimation between radiologists and between radiologists and AI

**Figure 1.**
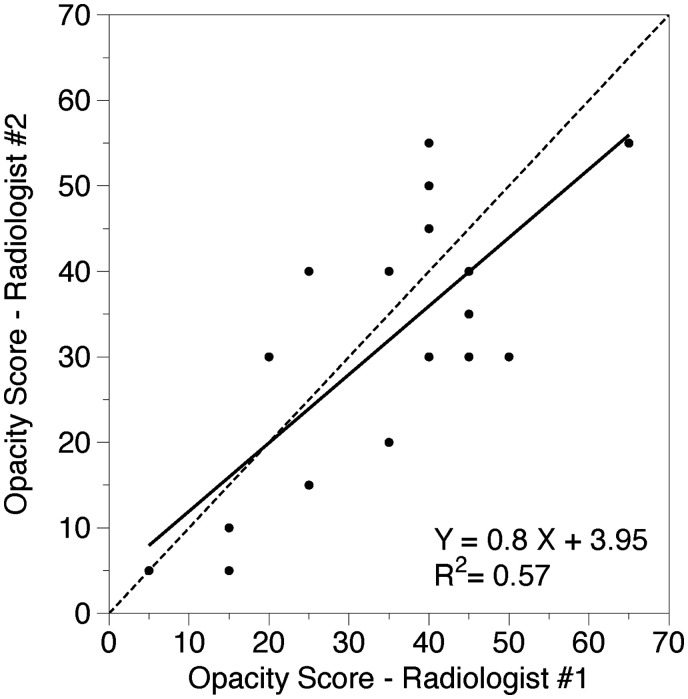
Correlation between 2 radiologists. The solid black line shows the linear regression analysis of the relationship between the opacity quantification at CTPA performed by 2 radiologists (%), dashed line shows the equality line.

**Figure 2.**
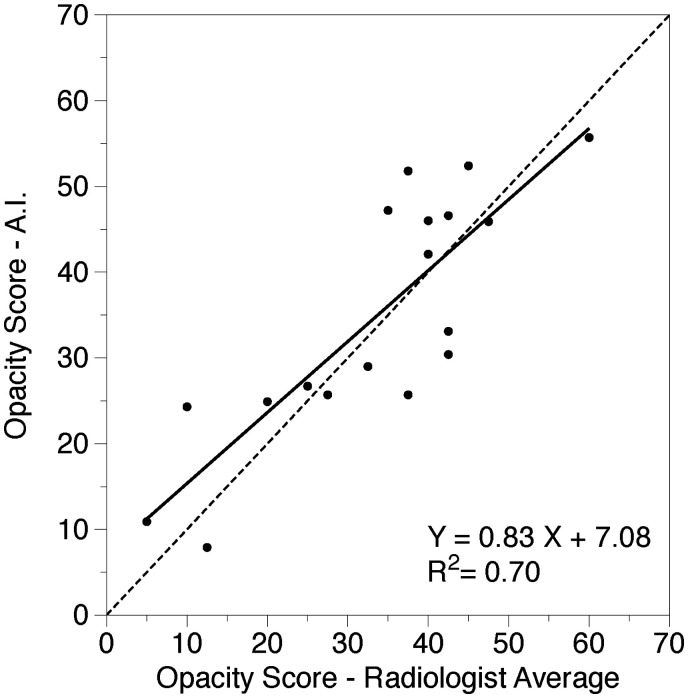
Correlation between radiologists and AI. The solid black line shows the linear regression analysis of the relationship between the average opacity quantification at CTPA performed by 2 radiologists versus that determined via the AI protocol (%), dashed line shows the equality line.

A strongly positive linear correlation was present between the 2 radiologists’ estimations (*r*^2^ = 0.57, *P* < .001) and between the AI data and the mean scores of the 2 radiologists (*r*^2^ = 0.70, *P* < .001) (Figures 1 and 2).

#### Bland-Altman plot analysis for lung opacity quantitative estimation between radiologists and between radiologists and AI

**Figure 3.**
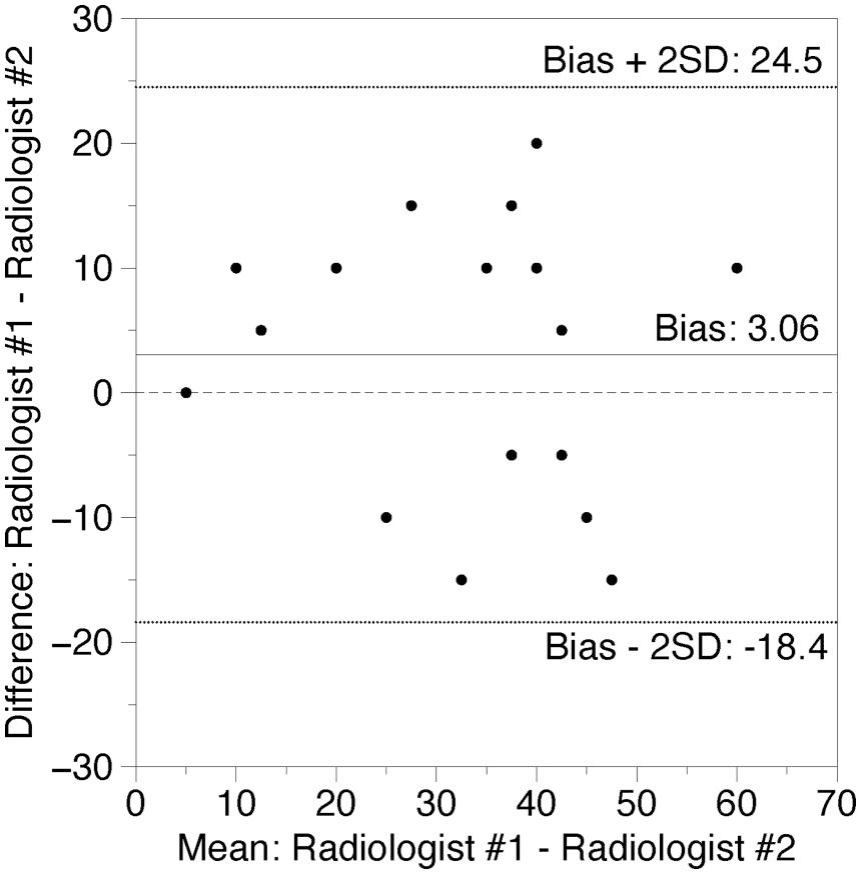
Bland-Altman relationship between 2 radiologists. The *x*-axis of the plot displays the average quantity of opacity measured by the 2 radiologists and the *y*-axis displays the difference in measurements between both measurements (*n* = 18). Data expressed in %.

**Figure 4.**
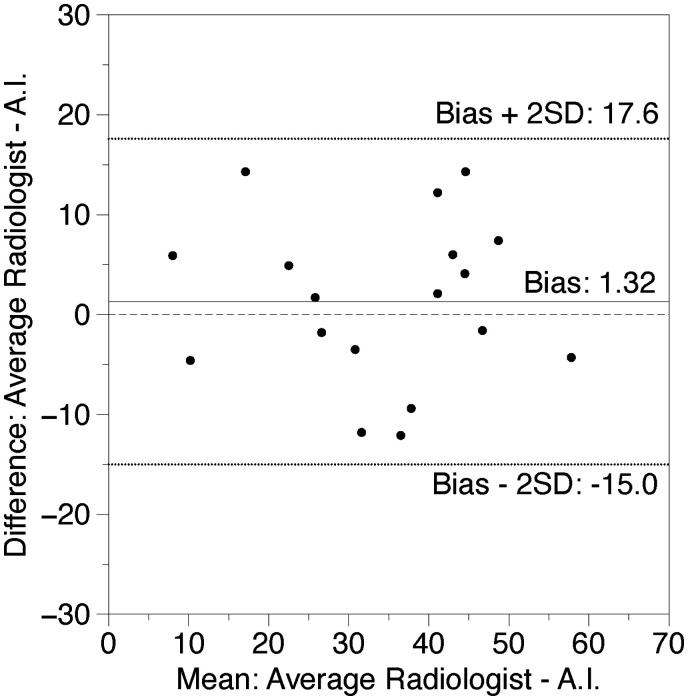
Bland-Altman relationship between radiologists and AI. The *x*-axis of the plot displays the average quantity of opacity measured by AI and the average of the 2 radiologists and the *y*-axis displays the difference in respective measurements (*n*=18). Data expressed in %.

Bland-Altman plots showed a mean difference of 3.06% between the 2 radiologists’ scores and −1.32% for the mean of the radiologist scores vs AI, respectively. No outliers were detected outside the range of 2×SD for both measurements (+24.51%: −18.4% and +14.98%: −17.62%, respectively). Data are shown in Figures 3 and 4.

#### ROC and AUC analysis of lung opacity quantification by radiologists and AI

**Figure 5.**
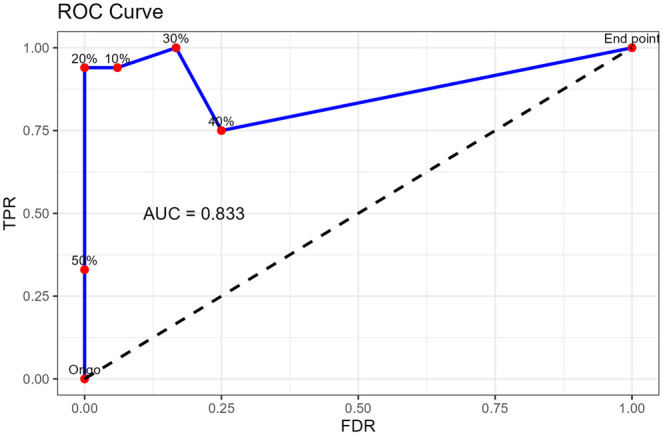
ROC and AUC curve analysis of the quantity of lung opacities measured by the radiologist and AI. Abbreviations: AUC = area under curve 0.83 indicates good system performance; TPR = true positive rate; FDR = false discovery rate.

The ROC curve is shown in Figure 5. The AI achieves its best performance at a threshold value of 20%, where sensitivity is high, and the false discovery rate is low. Beyond a threshold of 30%, the AI's ability to correctly identify true positives diminishes, as indicated by a decrease in sensitivity. An AUC value of 0.83 (CI 0.5−1) demonstrates the system's good overall accuracy. The sensitivity, specificity, and accuracy for the analysed thresholds are summarized in Appendix S3.

The analysis output from the AI-Rad Companion software is summarized in Appendix S4.

## Discussion

Our study explores the impact of radiological contrast on the accuracy of AI algorithms trained on non-contrast CT scans in quantifying lung opacities during the COVID-19 pandemic. The early pandemic posed major challenges for healthcare systems. At the radiology departments, swift decisions were made on how to best use diagnostic imaging, balancing the need for prompt and accurate diagnosis against the risks of healthcare system overload and potential exposure of healthcare workers. Therefore, initial guidelines advised against using CT for COVID-19 diagnosis due to concerns about healthcare system capacity.^17^ As the understanding of COVID-19 evolved, a high risk of PE in this patient group was identified, and CTPA became an acceptable exception for this indication. This approach raised new questions about whether contrast-enhanced CTPA images could affect the detection and quantification of lung opacities associated with SARS-CoV-2 infection.

### Lung CT and iodinated contrast medium

The most common indication for performing a contrast-enhanced CT scan of the chest involves diagnosing macroscopic vascular pathology and, in some cases, pleural diseases. In cases of suspected PE, CTPA is typically utilized. However, imaging changes in the airways/alveoli and interstitial space are crucial elements of the radiological diagnosis of acute respiratory diseases, and conducting additional images without contrast enhancement would result in unnecessary exposure of the patient to excessive X-ray radiation.^18^

The presence of radiological contrast agents used in CTPA can potentially contribute to the overall opacity level observed in the CTPA images. The effect of iodinated contrast medium on the attenuation and quantitative assessment of lung tissue has been the subject of research in patients with pulmonary emphysema, yielding divergent results. Heusell et al stated that contrast examination is not comparable to examination without contrast enhancement and shows falsely reduced amount of emphysema.^19^ However, Wiedbrauck et al, using an AI pulmonary density software similar to ours, reached the opposite conclusion and suggest only a small effect of contrast medium.^20^

In our cohort, a mean opacity of 34.8% was observed when assessed by AI, and 33.5% by the mean of both radiologists’ visual assessments. However, none of the patients in our study group was deemed ill enough to have been treated in the intensive care unit. Thus, our findings cannot necessarily be extended to severely ill patients with COVID-19. In a previous study involving patients with acute COVID-19, CTPA images analysed for an opacity count of <50% without PE were associated with no 30- and 60-day mortality compared to those with widespread (over 50%) parenchymal involvement and PE present.^10^

### AI-based quantitative lung opacity assessment and images without contrast enhancement of COVID-19 patients

Arru et al^9^ used the same CT pneumonia analysis tool in the assessment of the COVID-19 patient outcomes (death) and the need for ICU admission and concluded its superiority when compared to subjective assessments by the radiologist. Using the same deep learning algorithm, in a similar study, Homayounieh et al^13^ report a high correlation between quantitative analysis of observed lung lesions and qualitative subjective assessment of disease severity. Both studies however were performed on the larger cohorts of significantly more severe cases with high ICU admission rates and mortality, compared to our study group.

Manual quantitative calculations of the opacity scores are time-consuming and can suffer from low reproducibility.^21^ Utilizing the same AI tool as in our study, Turcato et al investigated the correlation between the quantified non-contrast–enhanced lung opacities and oxygenation and showed a higher percentage of lung involvement on CT in patients with the lower PaO_2_/FIO_2_ ratios.^22^ Xi et al compared the visual quantification of COVID-19 CT scans with another AI-based software, 3D Slicer, combined with manual segmentation, demonstrating its superiority in the context of clinical severity score.^23^ Lanza et al suggested the usefulness of AI-based CT analyses combined with manual segmentation in triaging of pandemic cases.^24^ Colombi et al used similar visual and software-assisted quantification of the extent of aerated lung and showed that both were equal predictors of intensive care unit admission or death.^25^

In another report utilizing dedicated CT analysis software, Liu et al demonstrated its superiority in predicting the clinical course, when compared with D-dimer and Acute Physiology and Chronic Health Evaluation II (APACHE-II) score.^26^

The use of automated AI enables precise and prompt annotation, but questions remain regarding the impact of the intravenous contrast agent on the opacity score. The AI algorithm utilized in this study was developed based on non-contrast–enhanced lung CT scans, enabling the quantification of various lung parenchymal changes of different attenuations. We did not know whether it would recognize intravascular opacities from contrast and (undesirably) include such in its reported overall assessment, or in contrast, whether it would “see” such opacities as non-parenchymal and (appropriately) exclude them.

The observed similarity in the results between radiologists (who sought to exclude contrast-based opacities) and AI suggests that the selected segmentation method effectively differentiates between the lung parenchyma areas and other structures imaged in the CT scans across both quantitative assessment methods. This likely indicates that the presence of contrast does not influence the total number of COVID-19 characteristic opacities measured, nor does it affect the diagnostic value of CTPA in this context.

Automated AI enables precise and rapid annotation but raises concerns about the impact of intravenous contrast on opacity scoring. The AI used in this study, developed for non-contrast–enhanced CT scans, quantifies lung parenchymal changes but was untested for contrast-enhanced scans. The similarity between radiologists’ results (excluding contrast-based opacities) and AI outputs suggests effective segmentation, indicating that contrast does not affect the quantification of COVID-19 opacities or the diagnostic utility of CTPA.

While AI tools are validated for non-contrast scans, their use in post-contrast settings requires further study. Variability in contrast dose, which affects HU values, remains a challenge. Integrating contrast injection data into DICOM files could enhance reliability by minimizing variability. This study provides foundational insights into AI’s potential in post-contrast imaging, supporting future research.

The absence of fibrosis or chronic sequelae in CT findings reflects the acute phase of COVID-19 in our patients, aligning with the expectation that such changes develop over time after prolonged inflammation. All reported findings are primarily associated with COVID-19, with minor atelectasis noted, and no unrelated sequelae identified. Extensive COVID-19 opacities may have obscured other potential findings, but the clinical context suggests a direct link to COVID-19.

### Study limitations

There are limitations to this study. Given the limited size of the studied cohort, validation in a larger sample is needed to confirm our findings.

The availability of AI tools for quantitative assessment of pulmonary lesions was limited to the one used in our study only.

A notable limitation and a possible bias of our study is the use of 2 different CT scanner models which introduced some protocol variability.

Gaining the opportunity for measuring the amount of lung opacities from the CTPA, with the use of non-contrast–enhanced scan-trained AI tools, can be a useful alternative to visual assessment. Our study findings reveal that for the evaluation of the quantity of COVID-19 lung opacities from CTPA images, AI tools trained on non-contrast–enhanced images demonstrate comparable results to visual assessments by experienced radiologists. Therefore, our article provides valuable preliminary information on the feasibility and potential of using AI in post-contrast settings, laying the groundwork for future advancements.

## Supplementary Material

tzaf008_Supplementary_Data
