# Supplementary material for: Quantitative assessment of lung opacities from CT of pulmonary artery imaging data in COVID-19 patients: artificial intelligence versus radiologist
Source: BJR Open. 2025 Apr 29;7(1):tzaf008. doi: 10.1093/bjro/tzaf008 (PMC12077292; doi:10.1093/bjro/tzaf008)
Supplement: tzaf008_Supplementary_Data [file tzaf008_Supplementary_Data.zip › BJR_oa_Appendix_III.pdf]

Appendix III.

ROC curve analysis at each threshold.

	50%	40%	30%	20%	10%
Sensitivity	0.333	0.75	1	0.9375	0.941
Specificity	1	0.80	0.75	1	0
Accuracy	0.889	0.778	0.889	0.944	0.889

The sensitivity, specificity and accuracy of the analysed thresholds of the amount of the lung opacities measured by the radiologists and AI.
