# Supplementary material for: Quantitative assessment of lung opacities from CT of pulmonary artery imaging data in COVID-19 patients: artificial intelligence versus radiologist
Source: BJR Open. 2025 Apr 29;7(1):tzaf008. doi: 10.1093/bjro/tzaf008 (PMC12077292; doi:10.1093/bjro/tzaf008)
Supplement: tzaf008_Supplementary_Data [file tzaf008_Supplementary_Data.zip › BJR_oa_Appendix_II.pdf]

Appendix II.

DESCRIPTIVE FINDINGS BEYOND LUNG OPACITIES IDENTIFIED BY RADIOLOGISTS			
	**Yes**	**No**	**Yes %**
**Findings**			
Ground-Glass Opacities (GGO)	18	0	100
Consolidations	17	1	94
Organizing Pneumonia (OP)	16	2	89
Crazy Paving pattern	8	10	44
Atelectasis	12	6	67
Bronchial Dilatation	9	9	50
Pleural effusion	3	15	17
Fibrosis	0	18	0
Nodules	0	18	0
Calcifications	0	18	0
Pulmonary Hemorrhages	0	18	0
**Distribution**			
Bilateral and Multilobar Involvement	17	1	94
Peripheral and/or Lower Zone Predominance	17	1	94

Descriptive findings of the lung opacities. Clarification of definitions: CT signs of Organizing Pneumonia (OP), as defined in the Glossary of Terms for Thoracic Imaging by the Fleischner Society, include peripheral or bronchocentric consolidation, air bronchograms, curvilinear perilobular opacities, nodular opacities, and the atoll sign. We included parenchymal bands in this definition, as they have been observed as sequelae of COVID-19-related pneumonia as well as in the context of organizing pneumonia. Fibrosis, defined as irreversible changes such as traction bronchiectasis, bronchiolectasis, architectural distortion, volume loss, and potentially honeycombing, which reflect connective tissue replacement within the lung parenchyma. Bronchial Dilatation: Used as recommended by the Fleischner Society, this term describes temporary or potentially reversible bronchial widening in the absence of established chronicity. Crazy Paving Pattern: Focal areas of ground-glass opacities with well-defined margins, within which septal thickening and intralobular reticular lines can be identified, resembling crazy paving stonework. This pattern is often seen in conditions such as pulmonary edema and may indicate more severe illness in the context of COVID-19 and is clinically associated with acute respiratory distress syndrome (ARDS). Nodule: a nodule is a circumscribed, typically, but not always, round opacity measuring less than or equal to 30 mm in average diameter. Nodules that are part of the perilobular pattern are registered as organizing pneumonia (OP) rather than as independent nodules to emphasize their association with the COVID-19 pattern and to avoid signaling other underlying conditions.
