# Supplementary figures and images for: Quantitative assessment of lung opacities from CT of pulmonary artery imaging data in COVID-19 patients: artificial intelligence versus radiologist
Source: BJR Open. 2025 Apr 29;7(1):tzaf008. doi: 10.1093/bjro/tzaf008 (PMC12077292; doi:10.1093/bjro/tzaf008)

## Appendix IV

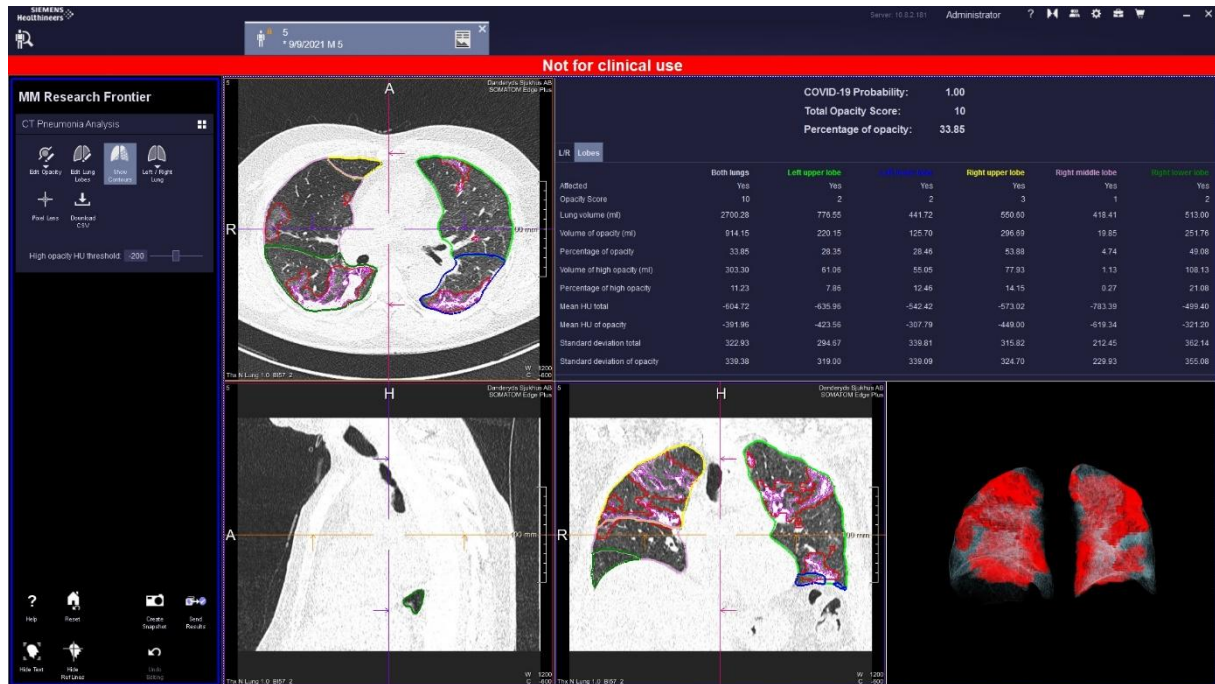

The analysis output from the AI Rad Companion software

Supplement: tzaf008_Supplementary_Data [file tzaf008_Supplementary_Data.zip › BJR_oa_Appendix_IV.pdf]
